# Conserved Expression Signatures between Medaka and Human Pigment Cell Tumors

**DOI:** 10.1371/journal.pone.0037880

**Published:** 2012-05-31

**Authors:** Manfred Schartl, Susanne Kneitz, Brigitta Wilde, Toni Wagner, Christiaan V. Henkel, Herman P. Spaink, Svenja Meierjohann

**Affiliations:** 1 Physiological Chemistry I, Biocenter, University of Würzburg, Würzburg, Germany; 2 ZF-Screens B.V., Leiden, The Netherlands; 3 Institute of Biology, Leiden University, Leiden, The Netherlands; Faculdade de Medicina, Universidade de São Paulo, Brazil

## Abstract

Aberrations in gene expression are a hallmark of cancer cells. Differential tumor-specific transcript levels of single genes or whole sets of genes may be critical for the neoplastic phenotype and important for therapeutic considerations or useful as biomarkers. As an approach to filter out such relevant expression differences from the plethora of changes noted in global expression profiling studies, we searched for changes of gene expression levels that are conserved. Transcriptomes from massive parallel sequencing of different types of melanoma from medaka were generated and compared to microarray datasets from zebrafish and human melanoma. This revealed molecular conservation at various levels between fish models and human tumors providing a useful strategy for identifying expression signatures strongly associated with disease phenotypes and uncovering new melanoma molecules.

## Introduction

Melanoma is one of the most aggressive forms of cancer with still rapidly increasing incidence in the western world [1] (http://seer.cancer.gov/csr/1975_2008/browse_csr.php?section=16&page=sect_16_table.05.html). Treatment opportunities arise from a large portfolio of candidate drugs some of which have made it to clinical studies; however, with differing and often unpredictable outcomes. Thus the need for a better molecular understanding of melanomagenesis and preclinical studies in-vitro and in animal models is undisputed [2].

Melanoma is a paradigm for the complexity of cancer. Melanomas arise from pigment cells of the skin, from extracutaneous sites and from the uvea of the eye. A certain fraction of cutaneous melanomas form on the basis of nevi, which then represent a precursor lesion. Others are supposed to originate from single pigment cells of the skin. The clinical heterogeneity of the disease is astonishingly high, ranging from spontaneous total remission to extremely fast, fatal progression. Although gene expression signatures of melanomas have been reported [3], [4], [5], [6], [7], only few clues were obtained for molecular subtypes that could be of clinical relevance. Obvious differences were more correlated to anatomical sites, treatment history of patients, and progression stage. A further complication widely discussed to camouflage a clear diagnostic gene expression signature, are individual genetic differences and recurrent changes that reflect epiphenomena of the transformed phenotype and the pathological physiology of the melanoma cells. In general, and especially in the melanoma field, high throughput transcriptome studies have so far not revealed the expected consensus alterations that would help to ultimately understand melanoma biology and pathology (for discussion see [8]. To pinpoint relevant expression patterns common to all tumor subtypes important information can be obtained from a cross-species comparative approach with melanoma animal models. Changes in gene expression that are conserved over large evolutionary distances have a high probability of reflecting common molecular mechanisms critical for the development of the same disease in different organisms [9], [10], [11].

We have developed a new model for pigment cell cancer in small laboratory fish [12] that cannot only be used for functional analyses but is also suited for high throughput studies. In this model the melanoma oncogene *xmrk* from *Xiphophorus* [13] is expressed under control of the *mitf* promoter in transgenic medaka fish. Medaka is a complementary model to zebrafish with similar characteristics and advantages for biomedical research [11]. Depending on a homogeneous, strain-specific genetic background, carriers of the transgene develop pigment cell tumors of different characteristics. These include uveal melanoma, exophytic epidermal pigment cell tumors of low malignancy, and invasive, metastatic melanoma. We used the new massively parallel sequencing technologies to establish transcriptomes of the different pigment cell tumor types and a precursor lesion and to provide a basis for comparison with human melanoma. We find in this animal model a high number of tumor-specific differentially regulated genes that have been assigned a diagnostic or functional role in human melanoma and we could identify sets of genes whose dysregulation is conserved in melanoma from fish to man.

## Results

For the RNA sequencing analyses three different tumor samples were used. These included a heavily melanized uveal melanoma (UM) that had already invaded the skull towards the central nervous system, a nodular, apparently exclusively exophytically growing xanthoerythrophoroma (XE) and an extracutaneous melanoma (MM), which was a large jet black tumor mass in the abdomen with massive invasion into the body musculature and metastasis to inner organs including the spinal cord. Heavily hyperpigmented skin (HP), a massive overproduction of pigment cells that are different in shape from the normal pigment cells that make up the basic pigmentation of the fish skin, but do not show any signs of three-dimensional growth or invasion, was used for comparison. Hyperpigmentation areas develop in fish only after initial activation of an oncogene. They have been called F-nevi and are regarded as the fish counterpart of human nevi [14].

At first the RNA-seq transcriptomes from the single tumors were analyzed. Of the 24662 ENSEMBL medaka transcripts between 9327 and 10376 transcripts were not expressed in the four transcriptomes. Comparing the invasive MM with the exophytic XE-tumor revealed 1442 transcripts that were only found in XE, while 1789 were detected only in MM. 238 transcripts were at least 10 fold (up to 42 fold) higher in XE than in MM, while 176 showed at least 10 fold (up to 72 fold) higher expression in MM than in XE (Table S4).

From all annotated medaka transcripts 18415 had been assigned a gene name. Only those (plus a few that we annotated ourselves) were considered further. Expression of several genes correlated with the different sublineages of pigment cells. As a specific feature of the fish model, pigment cell tumors can either be derived from melanin synthesizing cells or from pigment cells, which contain pteridins and carotinoids. Consequently, we found high expression of melanin pathway genes in MM and UM. The XE tumor and the hyperpigmented skin showed low expression of these genes, but abundantly expressed e.g. the rate limiting key enzyme of the pteridine pathway, guanylyl cyclohydrolase.

The fish pigment cell tumors showed for several established melanoma markers expression profiles comparable to mammalian melanoma, for instance high expression of MART1/MLANA [15], and upregulation of N-cadherin with simultaneous downregulation of E-cadherin [16]. The candidate suppressor of malignant melanoma AIM1 was downregulated as well [17]. Consistent with earlier findings in fish [12], mouse and human melanoma [18], [19] the pigment cell specific transcription factor *mitf*, which has been assigned a key role in maintaining the proliferative state of melanoma cells [20], [21], [22], was upregulated.

A number of regulators of cell proliferation were differentially expressed. CyclinD1, which is overexpressed in several neoplasms and amplified in subset of melanomas [23] was higher expressed in the tumors. While expression levels of the retinoblastoma gene RB1 were unchanged, a strong downregulation of all members of the p53 tumor suppressor gene family was observed.

The inhibitor of apoptosis BCL2 was considerably upregulated, accompanied by a slight downregulation of FAS and higher expression of FAS apoptotic inhibitory molecules1 and 2.

Growth factors and growth factor receptor signaling are very important for various aspects of the malignant phenotype of melanoma [24]. Of the many changes that we observed only some can be mentioned here, for instance a 5 to 10 fold upregulation of KIT and more than 15 fold upregulation of one of the two ERBB3 paralogs in MM and UM paralleled by a strong decrease of melanocortin receptor 1. From the SRC kinases only FYN was higher expressed in the tumors compared to the benign precursor lesion, consistent with its more prominent role in *xmrk*-driven melanoma [25], [26].

Members of the RAS and RAF families, in particular N-RAS and B-RAF, have attracted a lot of attention because they were found to be mutated in a majority of human melanoma [27] but transcript levels are generally not changed (https://www.oncomine.org). Accordingly, neither in the RNASeq transcriptomes nor in a larger survey on single fish tumors, regulation of *ras* or *raf* genes was seen (data not shown).

Reactive oxygen species (ROS) metabolism is an important regulator of oncogene-induced senescence and could be instrumental in the switch of benign precursor lesions to malignant tumors [28], [29]. ROS detoxifying enzymes (e.g. catalase, peroxiredoxin 6, superoxide dismutase) were considerably upregulated in all tumor types. This is in accordance with proteome data from *Xiphophorus*, where an increase of peroxiredoxins and other ROS metabolizing enzymes was observed during melanoma development [30], [31].

The *myc* genes are central players in the development of many cancers, but little is known about their role in melanoma. We found upregulation of *c-myc, N-myc* and of the c-MYC target and cell cycle progression activating transcription factor FOXM1.

An important feature of melanoma progression is that the switch from proliferative to invasive phenotype correlates with changes in wnt/ß-catenin signaling. In the tumors all Wnt family ligands were downregulated. In the m ore aggressive MM and UM there was a concomitant strong upregulation of the Wnt signaling antagonists *dickkopf 3* and *secreted frizzled-related protein (frzB)*, while in the XE tumor only a slight upregulation of *frzB* was noted.

**Figure 1:**
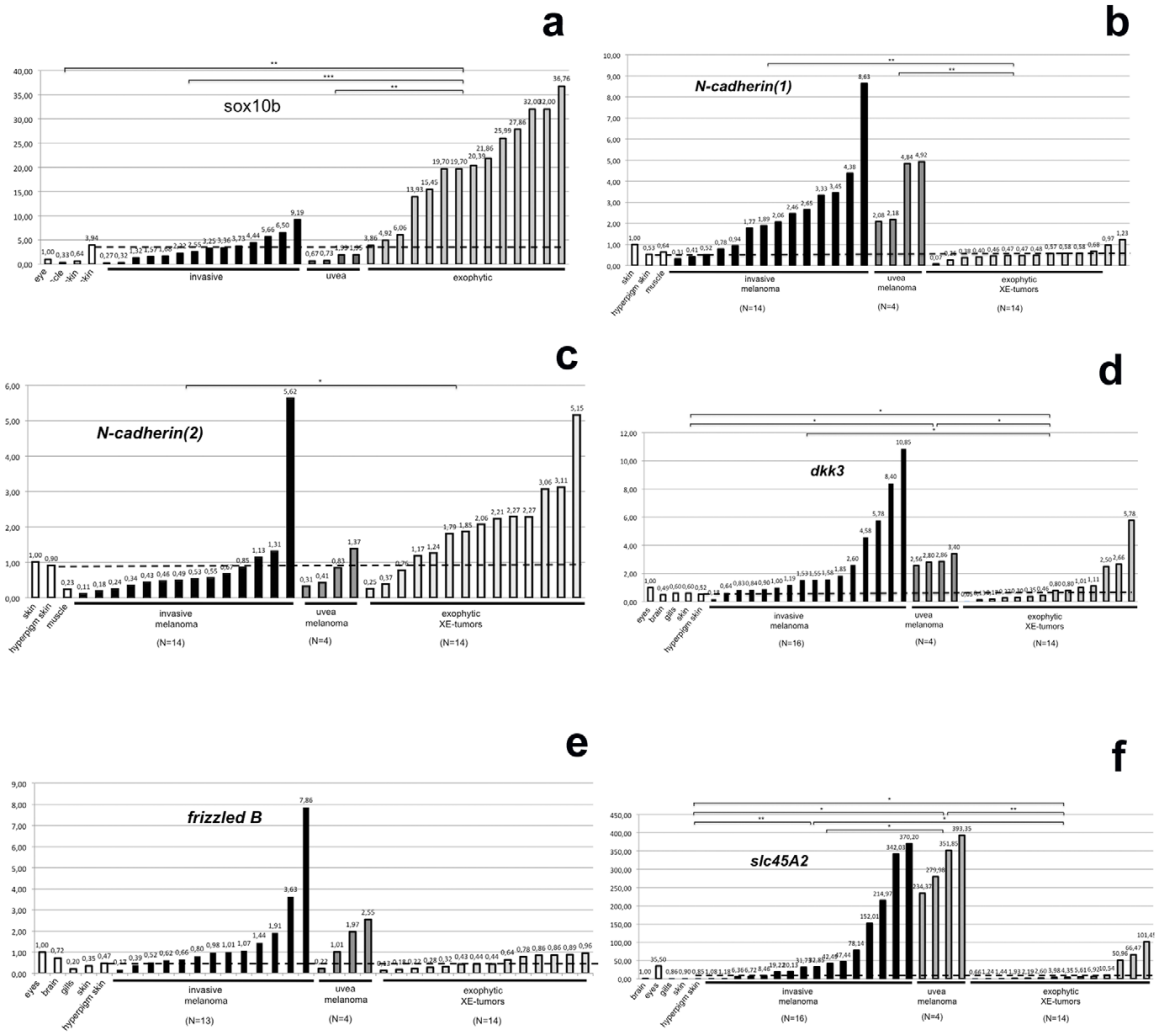
Quantitative real-time RT-PCR expression analyses of single MM, XE and UM tumors and hyperpigmented skin. 1a: sox10b expression; 1b: N-cadherin expression (variant 1), 1c: N-cadherin expression (variant 2), 1d: dkk3 expression, 1e: frizzledB expression. 1f: slc45A2 expression, notably the three most highly expressing XE tumors (to the right) had some black pigmented areas. ef1-alpha served as reference. For comparison, gene expression in normal tissue is shown. Groups showing significant differences in their expression are marked with *. Kruskal-Wallis p-value for frizzledB was 0.051 and therefore only slightly above significance level.

To validate the RNASeq data that were generated from one tumor each quantitative RT-PCR of a larger series of single tumors was performed and compared to expression in healthy organs, including skin, and the precursor lesion. In most cases expression differences seen in the transcriptomes were also obtained for a larger number of individual tumors. Of 22 arbitrarily selected genes, 14 showed the expression profile predicted from RNA-seq, 6 were in the range of the transcriptome data with single tumors of the whole set where expression was not in line with the RNA-Seq, while in two cases the transcriptome data were not confirmed. Generally, genes were expressed in each tumor type with some variation over all samples resulting in a continuum of expression levels (Figure 1). For genes, which in fish due to the teleost-specific whole genome duplication are present in two isoforms, we frequently noted a tumor subtype specific expression (for example *N-cadherin*; Figure 1b, c).

An important feature of RNAseq transcriptome data is that they provide information on alternatively spliced mRNAs. Of all genes (n = 3151) that are annotated with different mRNA isoforms 614 were not expressed. 1776 genes were expressed as a single transcript. 196 showed differential regulation and 565 displayed regulation in the same direction. Of note, one of the three isoforms of *mitfa* was highly upregulated in XE and MM, while a different transcript was upregulated in UM (Table S3).

The above-indicated changes were all detected by screening the RNAseq dataset for known players of tumor development. To obtain information on a more systemic level we attempted to make use of algorithms for the analysis of microarray data. Plotting the average signal intensity of all groups versus fold change between mean tumor log2 values and log2 of hyperpigmented skin for each gene, 85% of the genes display a less than 4-fold regulation with an average log ratio of zero indicating that there is no bias to either up or down regulation. Given a threshold for regulation of logFC >2, p-value <0.05 and provided that a gene is expressed (base mean >10) in at least one group, in a comparison of the tumors to each other or to hyperpigmented skin we found 640 genes to be commonly regulated. However, differential expression could be easily extracted on the single gene levels for the apparent outliers of the MA-plot (Figure S1). For instance (Figure S2), t*yrp1* is highly up regulated in MM and UM. GCH1 is absent from UM, but high in the three other samples. CYTL1 is highly expressed in HP, though almost not expressed in all three tumor types. Conversely, MLANA is very high in all tumors but absent in the premalignant HP.

**Figure 2:**
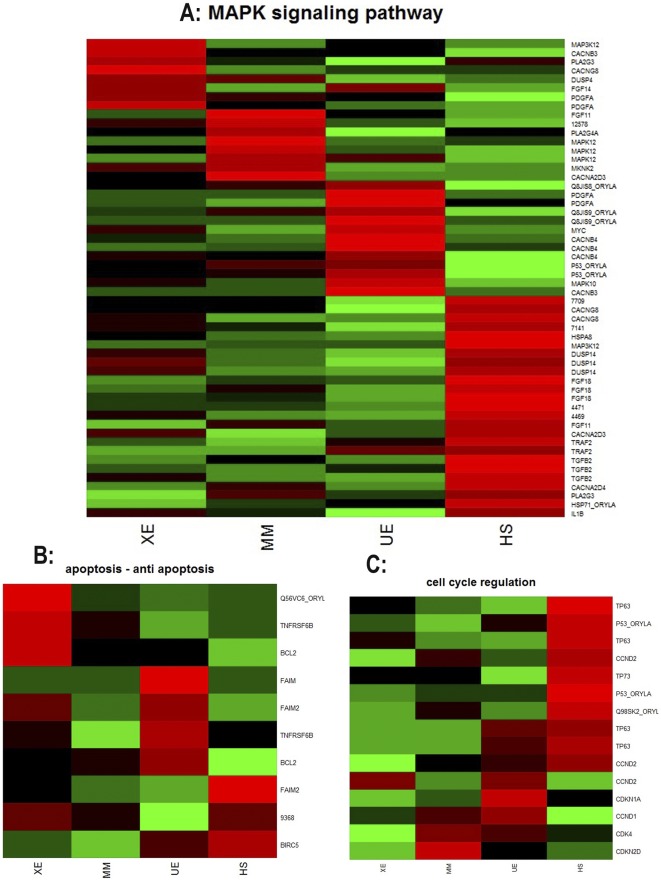
Heatplot (scaled) of differentially expressed genes from the MAP kinase pathway (2a), apoptosis (2b) and cell cycle regulation (2c), displaying the quality of the read count distribution within the genes. Low read counts are colored in green, high read counts are colored in red.

**Figure 3:**
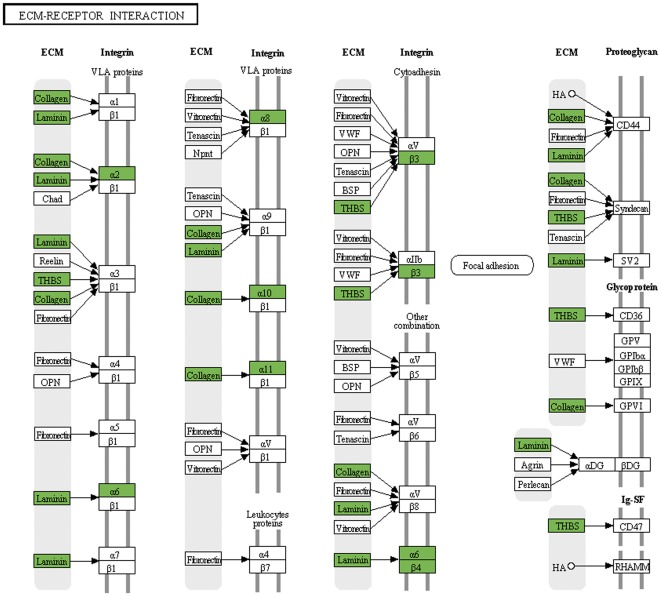
Regulation of genes involved in ECM-receptor interaction (KEGG pathway: 04512). Genes displaying a logFC>2 and p-value<0.05 in all three tumor types compared to hyperpigmented skin are marked green (down-regulated) squares.

Using the functional annotation tool of the DAVID bioinformatics resource revealed significant regulatory changes of certain pathways. Each tumor was characterized by a unique set of regulated genes but there were also common regulatory changes when all three tumors where compared to HP (Table S2). Not unexpectedly, coordinated regulation of genes from major metabolic pathways (glycolysis, pentosesphosphate pathway, metabolism of aminoacids and nucleotides), cell cycle control and MAP kinase pathway within samples but different for each tumor type became apparent (Figure 2). UM showed a more pronounced upregulation of the protein translation machinery than MM and XE. Consistently in all tumors there was downregulation of genes involved in extracellular matrix (ECM) – receptor interactions, cell adhesion molecules and cellular junctions (Figure 3, Figure S5). Downregulation of cell adhesion molecules was up to 10 fold stronger in invasive tumors than in non-invasive. In all affected pathways, a remarkably high number of pathway components were altered, e.g. of the 67 ECM-receptor interactions listed in the KEGG pathway 54 showed a regulatory change in at least one component. 40 showed a downregulation in all tumor types. All comparisons consistently revealed changes in regulators of the calcium-signaling pathway.

Transformation of the RNA-Seq data also allowed comparison of our data set to microarray expression analyses from zebrafish melanoma [32]. Except for RAP2B all genes upregulated in zebrafish were also upregulated in at least one tumor type in medaka (Figure S3). In case of downregulated genes the majority of the medaka genes (47 of 63, including different transcripts of the same gene) behaved similarly as their orthologs in zebrafish melanoma. Genes from the common cross-species expression signature include pigment cell specific genes like tyrosinase but also more widely expressed regulators of intracellular signaling transduction, e.g. sprouty4.

Finally, we wanted to determine regulatory changes that are conserved between pigment cell tumors in the fish model and human melanoma. From the Talantov microarray datasets from human samples 18 benign melanocytic skin nevi and 19 cutaneous primary melanoma [33] were used, which appeared to be most appropriate for comparison to our dataset, aligning the fish nevi (HP) to human nevi and all fish tumors (XE,MM,UM, combined into one group) to cutaneous primary melanoma. The analyses revealed 49 genes that were commonly downregulated in fish and human tumors while only MAP3K12 was found to be commonly upregulated. Comparing the data from the medaka MM to the human dataset revealed 65 genes to be commonly downregulated and 20 upregulated genes. Assigning those genes to defined pathways (Figure S4) uncovered genes involved in focal adhesion, cell adhesion, ECM-receptor interaction, and markers of neuroectodermal cells.

**Figure 4:**
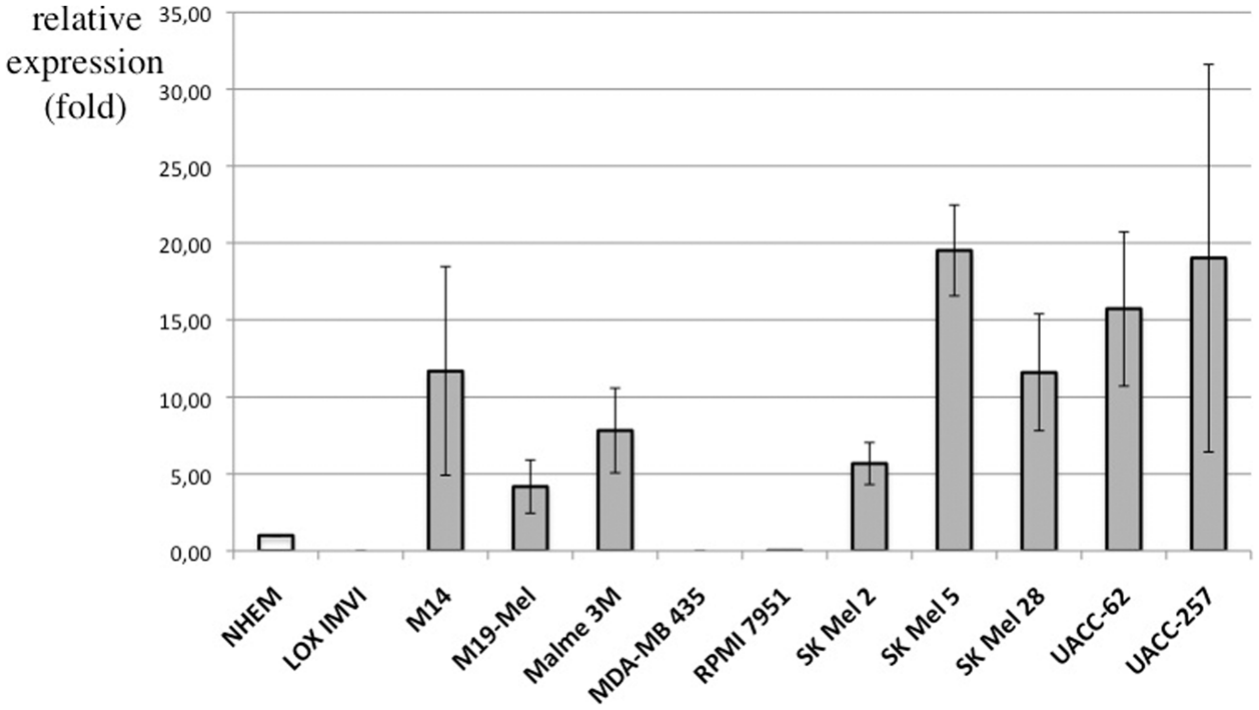
Quantitative real-time RT-PCR analysis of *SLC45A2* expression in LOX IMVI, M14, M19-Mel, Malme 3M, MDA-MB 435, RPMI 7951, SK Mel 2, SK Mel 5, SK Mel 28, UACC-62, and UACC-257 melanoma cells. NHEM cells served as expression control and were set as 1. beta-actin was used as reference gene.

We also compared the fish tumor transcriptomes to the 105 genes that are derived from a large metaanalyses of microarray data from human melanoma cells that has basically defined two main motifs [34]. Of the 38 genes common with medaka from motif 1, which is expressed by proliferative melanoma cells, 27 were upregulated more than 2 fold in all tumors. From motif 2, which characterizes the invasive and prometastatic phenotype, 34 genes were shared with medaka, of which 27 were upregulated more than 2 fold (Table S5):

To evaluate novel potential markers derived from our comparative analyses, the entire set of melanoma cell lines from the NCI60 panel was tested for expression of SLC45A2. This revealed that in comparison to normal human epidermal melanocytes (NHEM) eight of eleven melanoma cell lines showed considerably enhanced expression levels of this gene (Figure 4). SLC45A2 is a known component of the melanin pathway, but in the tumors it was found to be upregulated even though they were not melanized.

## Discussion

RNA sequencing has so far not been used in fish models to detect global expression changes that are helpful for better understanding processes of tumor growth and progression. We show that the enormous power of the new sequencing technology can be combined with the already existing wealth of data from chip-based microarray data. We did not search for possible mutations, gene fusion transcripts and novel transcripts, because in our melanoma model (like in other transgenic or natural genetic models for cancer) the primary cause for tumor development is known, here contributed by the specific overexpression of an activated oncogene. In addition the short latency period of melanoma development makes additional sporadic genomic alterations and random mutations very unlikely to be critical for tumor initiation. However, in other situations, where the causative event of melanoma is unknown, RNA sequencing can be useful also for detecting genomic changes relevant to tumor formation ([7]).

As a starting point for our analyses we used the transgenic *mitf::xmrk* medaka melanoma model, which like transgenic mouse models provides the advantage that the primary oncogenic trigger for tumorigenesis is always the same. Due to the defined genetic background melanoma development is stereotypic and the research material should be comparable from individual to individual. Importantly, our analysis shows that it is essential to verify data by single gene/single tumor analysis, using quantitative RT-PCR. Even in our melanoma model system with a more uniform genetic background than the human population we found an unexpected variation between samples. Certainly, melanoma classification in fish is much less elaborate than in humans, but special care was taken to collect tumors with comparable growth characteristics, tumor size and location. This indicates that even on a common genetic basis every single melanoma develops its own molecular expression program, despite similar disease phenotypes.

Small aquarium fish have become well accepted and useful models for melanoma research [11] and have shown great promise for drug discovery and detecting new melanoma genes [32], [35]. Previous studies have used these systems as surrogates for analyzing the action of mutated oncogenes in eliciting melanoma formation and the interaction of known melanoma pathways with the primary oncogene. One study compared the expression profile of 16 candidate genes for cancer progression in a mutant HRAS transgenic zebrafish melanoma model with data from human melanoma, but found no consistent expression changes [36]. Another study reported microarray analyses of melanoma in mutant NRAS;p53−/− zebrafish in comparison to normal skin and human melanoma [37]. These authors noted “a high degree of molecular similarity” of fish and human disease. However, only up-regulated genes were found to be conserved between zebrafish and human melanoma. As normal melanocytes are only a minor constituent of normal skin (used as the reference tissue) downregulation of genes was rather difficult to deduce. We used hyperpigmented skin for control where pigment cells make up a major fraction and readily detected a number of conserved downregulated genes.

For the BRAF;p53−/− zebrafish model a microarray gene expression profile from melanoma was compared to embryonic stages. Gene enrichment analyses uncovered a signature of 123 overlapping genes, which is similar to the signature of multipotent neural crest progenitors [32]. Comparison to this signature revealed a very good overlap with the gene expression profile of the medaka pigment cell tumors.

Recently a critical evaluation of the existing human melanoma microarray data was performed and uncovered strong discordance due to inhomogeneity of patient cohorts and tumor samples. From datasets showing the best match of material a meta-analysis was done [38]. This revealed a list of only 17 dysregulated genes that appear to be associated with melanoma progression. Interestingly, this includes many genes that we found commonly regulated between fish and human melanoma (e.g. BCL2, WNT family members) or which became evident in the fish tumor comparisons, e.g. components of the ECM, cell cycle regulators, PLP1, and CLIC3.

The profile of the fish pigment cell tumors showed also a large overlap with the motifs of gene expressions that were established from an extensive microarray analysis of many human melanoma cell lines [34]. However, the tumor with the more invasive and metastastic (MM) and the tumor with the more exophytic and less invasive growing (XE) showed no clear separation into the proliferative and invasive signatures. This may be explained by the fact that our data are from whole tumor biopsies. Even the exophytic tumor has areas of local invasion into deeper layers and the underlying body musculature and the MM has large areas of nodular growth. Thus, it can be expected that cells of both characteristic will contribute to the RNA pool extracted from the tumor.

The high expression of classical melanoma markers, upregulation of N-cadherin, downregulation of E-cadherin, inhibitors of cell cycle, growth promoting genes and inhibitors of apoptosis demonstrates that the fish melanoma share many common features on the gene expression levels equivalent to mammalian pigment cell tumors, thus providing useful models. An interesting aspect is the consistent downregulation of p53, p63 and p73. On the one hand, this is also a strong contribution to inhibition of apoptosis. On the other hand, the suppression of the p53/Cdkn2a arm of cell cycle control is know to be an important step in melanomagenesis, although mutations in p53 gene family members are generally more rare than in other tumors [39]. Repression on the transcriptional level of those genes is another way to produce a loss of function and could generate in a similar way an uncontrolled proliferation response [40]. The observed upregulation of cyclinD1 may lead in a similar way to the inactivation of the RB1-pathway of cell cycle control. A cooperative action of p53 loss for initial melanoma formation in zebrafish Braf and Nras models [14], [37] and for melanoma progression in the medaka *xmrk* melanoma model [12] has been documented.

The exact role of Wnt/ß-catenin signaling in melanoma is still controversial, although evidence has been presented that activation of Wnt/ß-catenin results in decreased proliferation and leads to upregulation of melanocyte differentiation genes [41]. The consistent downregulation of Wnt signaling components and the upregulation of Wnt antagonists in the more malignant and faster growing tumors is in line with the predominant proliferative nature of the tumors analyzed here. A deactivation of Wnt signaling was also seen in human melanoma showing the expression profile of highly aggressive and metastatic tumors [34].

Altogether our comparison of fish and human melanoma defines a highly conserved expression program of pigment cell tumors. It will be worthwhile to look in more detail into theses genes for their usefulness as melanoma biomarkers and a functional role for the malignant phenotype.

As a first example we found that the melanosome component SLC45A2 was upregulated in MM and UM. This was confirmed using the NCI60 melanoma cell lines. Interestingly this gene is also highly expressed in the malignant sample fraction of other human melanoma microarrays (https://www.oncomine.org). Hence it is a promising candidate for a melanoma marker. SLC45A2 has earlier been associated with melanoma only in the context of pigmentation, where mutations in the gene confer higher melanoma risk [42], [43].

## Materials and Methods

### RNA sequencing

Total RNA was isolated from a single uveal melanoma, exophytic xanthoerythrophoroma and invasive, metastatic melanoma, while hyperpigmented skin was pooled from 5 siblings (for detailed description of the genotypes and histopathology see [12] and extracted with miRNAeasy kit (Qiagen). RNA quality was checked using Agilent Bioanalyzer 2100 total RNA Nano series II chip. Transcriptome libraries were prepared from total RNA using Illumina mRNA-Seq Sample Preparation Kit. Libraries were sequenced to a single read length of 51 nucleotides on an Illumina GAIIx instrument according to the manufacturer's recommendations. This protocol does not yield miRNA sequences and does not allow fusion transcript identification. Image analysis and basecalling were performed using the standard Illumina pipeline. Resulting reads were trimmed of low quality nucleotides and aligned against 24662 cDNA sequences predicted by ENSEMBL's *O. latipes* Genebuild (version 56, www.ensembl.org) using the CLC bio Genomics Workbench version 3.6.5 (CLC bio, Aarhus, Denmark). Alignment counts were normalized for transcript length and total aligned reads (RPKM values) [44].

### Bioinformatic analyses

A threshold level for RPKM values to reflect gene expression above background was set to 2.

For easy and efficient comparison of transcriptomes we employed CrossQuery [45]. It uses a MySQL database backend with prejoined data-tables, which allows very fast query-returns. The RNASeq datasets were logically associated, mathematically filtered and sorted.

All data were analyzed using different R packages from the Bioconductor project (www.bioconductor.org). RNA sequence data were analyzed with “DESeq” [46], an R-package, which was written to test for differential expression in sequencing data. Briefly, based on the aligned count data size factors were estimated, which were used to calculate the effective library size. Count variance was estimated across conditions, based on the assumption that the majority of genes behave the same across conditions and the variation calculated for one condition would rather be too high than too low. This estimation of variance allows using single data sets as well as data sets having replicates. To detect differential expression, signal intensities, log-fold changes (logFC) and p-values were calculated for each gene. For the comparison of the fish RNA-Seq data genes were considered to be differentially expressed, if logFC>2 in case of single sample comparisons. For the comparison of all tumor types combined versus hyperpigemented skin, to ensure good comparability between tumor samples, in addition to a threshold for logFC the p-value was required to be less than 0.05.

Human and zebrafish orthologues of medaka genes were found by ENSEMBL IDs using “biomaRt”. Functional interpretation and clustering was done using the web-based annotation tool DAVID (http://david.abcc.ncifcrf.gov/), applying 0.01 as EASE threshold. For color display of pathways the KEGG Mapper (http://www.genome.jp/kegg/tool/color_pathway.html) was used.

For comparison with human tumor samples we selected Affymetrix microarray datasets from malignant melanoma and benign nevi [33], (GEO acc.no: GSE3189). Raw data from .cel files were quantile-quantile normalized [47], logFC and p-values were calculated based on a modified t-test using the limma package [48]. A gene was considered to be differentially regulated, having a fold change >2 and a p-value<0.05.

To obtain a common dataset for human and fish, logFC resulting from limma (human) and DESeq (medaka) were combined on the basis of their gene symbols. Only genes common to both datasets (n = 8289) were considered.

To obtain a set of alternatively spliced genes all gene IDs corresponding to more than one transcript ID were filtered out. Genes that were not expressed all tumor types (RPKM<2) were excluded. To detect different regulation between transcripts for each group and each gene (G_i_) mean RPKM of all transcripts (T_ij_) were calculated:
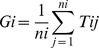
Correlations of each transcript to its corresponding gene mean values were computed and the range of the correlation values within each gene (RC_gene_) calculated. A gene was defined as alternatively spliced, if RC_gene_>1.

To relate the fish expression profiles in relation to the 105 genes defined for human melanoma cell phenotype-specific expression [34], (http://www.dermatologie.usz.ch/Research/hoek/information/Seiten/work_105.aspx) genes found to be differentially expressed in XE vs. HP or MM vs. HP resulting from the DESeq package were compared to those expressed by proliferative phenotype melanoma cells (motif 1) and genes expressed by invasive phenotype melanoma cells (motif 2).

### Cell culture

Human melanoma cell lines from the NCI-60 panel (LOX IMVI, M14, M19-Mel, Malme 3 M, MDA-MB 435, RPMI 7951, SK Mel 2, SK Mel 5, SK Mel 28,UACC-62, UACC-257) were cultivated in DMEM supplemented with 10% FCS, penicillin (100 U/ml, Gibco), and streptomycin (100 µg/ml, Gibco). The source of these human melanoma cell lines is the DCTD Tumor Repository, National Cancer Institute at Frederick, Frederick, Maryland. NHEM cells were from Promocell and kept in Melanocyte Growth Medium.

### Quantitative real-time PCR

RNA was extracted using TRIZOL (Invitrogen) or Total RNA Isolation Reagent (ABgene). After DNase treatment, reverse transcription was performed using Superscript II Reverse Transcriptase (Invitrogen) or RevertAid First Strand Synthesis kit (Fermentas) and random hexamer primers. cDNA from 15 ng of total RNA for *ef1* and 50 ng for all other transcripts was used for realtime PCR (for primer sequences see Table S1) using SYBR Green. Amplification was monitored with i-Cycler (Bio-Rad). All results are averages of at least two independent reverse transcription reactions and 2–5 PCR experiments from each such reaction. For quantification data were analyzed by the ΔCt method [49], and normalized to *ef1α* mRNA for medaka and to beta-actin for human samples. For spot check, not reversely transcribed RNA was used in control PCR reaction. Data are presented as mean ± standard deviation. Changes in mRNA expression were tested using a Kruskal-Wallis test and Mann-Whitney test, as post-hoc we used an approach based upon the Tukey method as described [50].

## Supporting Information

Figure S1 **MA-plot of all melanoma samples compared to hyperpigmented skin.** Average expression of all groups is plotted against the x-axis; average change of expression (log fold change) is plotted against the y-axis. Red spots indicating genes that have a p-value<0.01 and differential expression >4-fold up, green spots indicating genes that have a p-value<0.01 and differential expression >4-fold down in melanoma, grey spots indicate genes that were defined as not expressed. 12 genes showing the highest up-/down-regulation are annotated. Numbers represent the end digits of the respective Ensembl transcript ID.
(PDF)

Figure S2 **Log2 RPKM values of single differentially regulated genes in the tumors (XE, MM, UM) and hyperpigmented skin (HP).** TYRP1 (2of2), tyrosinase related protein 1, isoform 2; GCH1, guanylylcyclohydrolase 1; CYTL1, cytokine-like 1, isoform 2; MLANA, melan-A.
(TIF)

Figure S3 **Heatplot (scaled) of genes common in zebrafish and medaka, displaying the quality of the log2 read count distribution within the genes.** Low read counts are colored in green, high read counts are colored in red. 3a: Genes down-regulated in zebrafish, 3b: Genes up-regulated in zebrafish.
(TIF)

Figure S4 **Affected pathways based on genes commonly upregulated or downregulated more than 2-fold in human cutaneous primary melanoma compared to melanocytic skin nevi and fish tumors (XE, UM and MM) compared to fish nevi (HP).** Red bars show the number of observed genes up-regulated or down-regulated in the dataset, blue bars show the statistically expected number of genes, given the result to be random.
(PDF)

Figure S5 **Gene Ontology analysis of functional gene groups commonly regulated in medaka tumors versus HP.** The analysis was performed using the Gene Set Analysis Toolkit V2 (http://bioinfo.vanderbilt.edu/webgestalt/).
(GIF)

Table S1 **Primers used for quantitative real-time PCR analysis.**
(DOC)

Table S2 **List of genes with a more than 4-fold regulation in all tumors compared to the benign precursor lesion.**
(XLS)

Table S3 **List of differentially spliced and differentially expressed genes.**
(XLS)

Table S4 **Number of genes with RPKM>2 showing an at least 2-fold up or down regulation in different tumor types compared to hyperpigmented skin.**
(DOC)

Table S5 **Genes common in medaka tumor transcriptomes and the Hoek human melanoma gene expression signature.** Columns 5 to 8 indicate in which datasets a gene is commonly regulated (1) or not (0).
(XLS)
