# Supplementary figures and images for: Conserved Expression Signatures between Medaka and Human Pigment Cell Tumors
Source: PLoS One. 2012 May 31;7(5):e37880. doi: 10.1371/journal.pone.0037880 (PMC3365055; doi:10.1371/journal.pone.0037880)

**p.value < 0.05, logFC > 2**

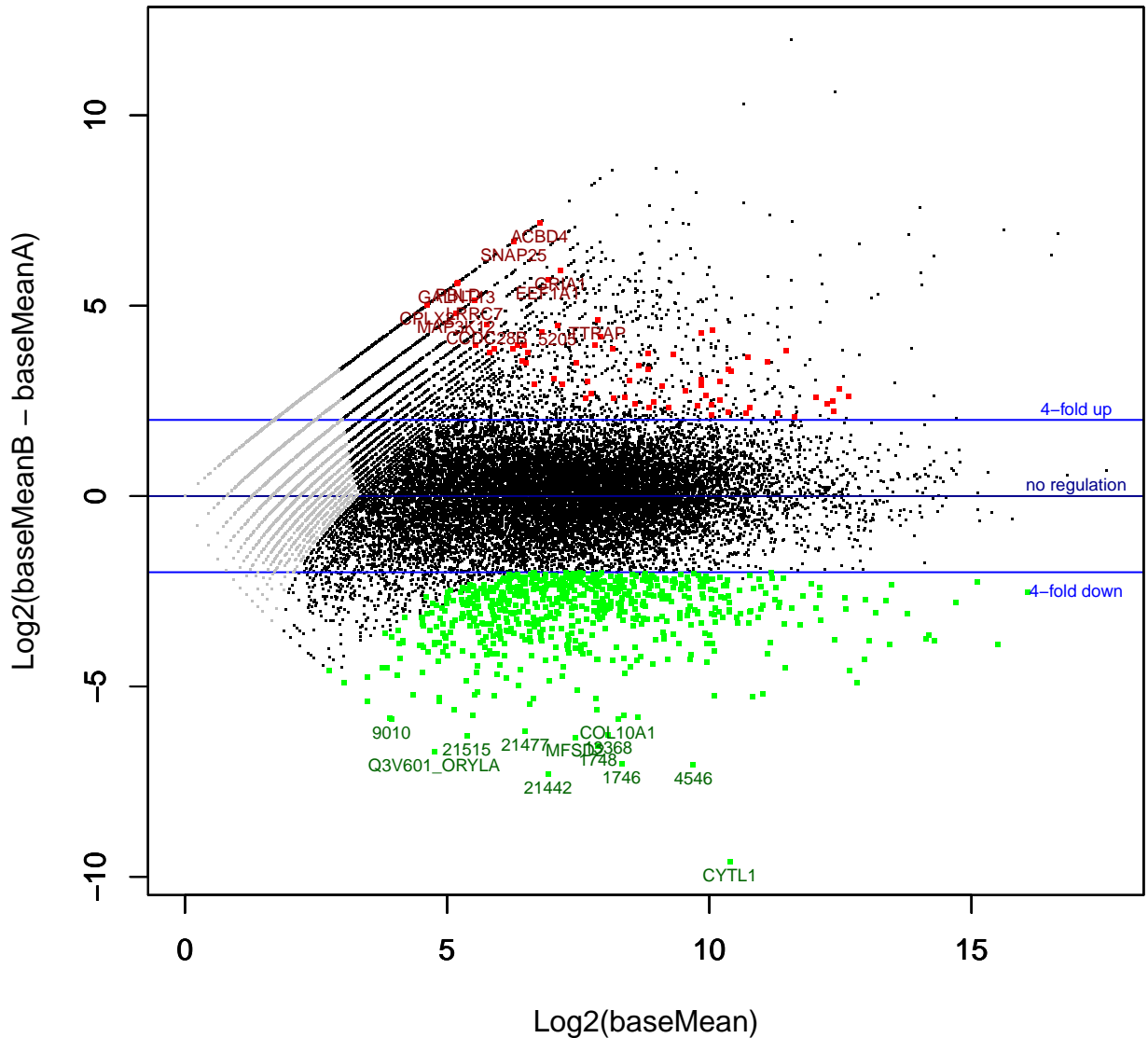

Supplement: Figure S1 — MA-plot of all melanoma samples compared to hyperpigmented skin. Average expression of all groups is plotted against the x-axis; average change of expression (log fold change) is plotted against the y-axis. Red spots indicating genes that have a p-value<0.01 and differential expression >4-fold up, green spots indicating genes that have a p-value<0.01 and differential expression >4-fold down in melanoma, grey spots indicate genes that were defined as not expressed. 12 genes showing the highest up-/down-regulation are annotated. Numbers represent the end digits of the respective Ensembl transcript ID. (PDF) [file pone.0037880.s001.pdf]

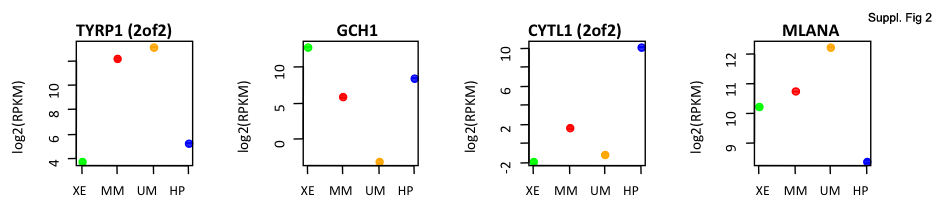

Supplement: Figure S2 — Log2 RPKM values of single differentially regulated genes in the tumors (XE, MM, UM) and hyperpigmented skin (HP). TYRP1 (2of2), tyrosinase related protein 1, isoform 2; GCH1, guanylylcyclohydrolase 1; CYTL1, cytokine-like 1, isoform 2; MLANA, melan-A. (TIF) [file pone.0037880.s002.tif]

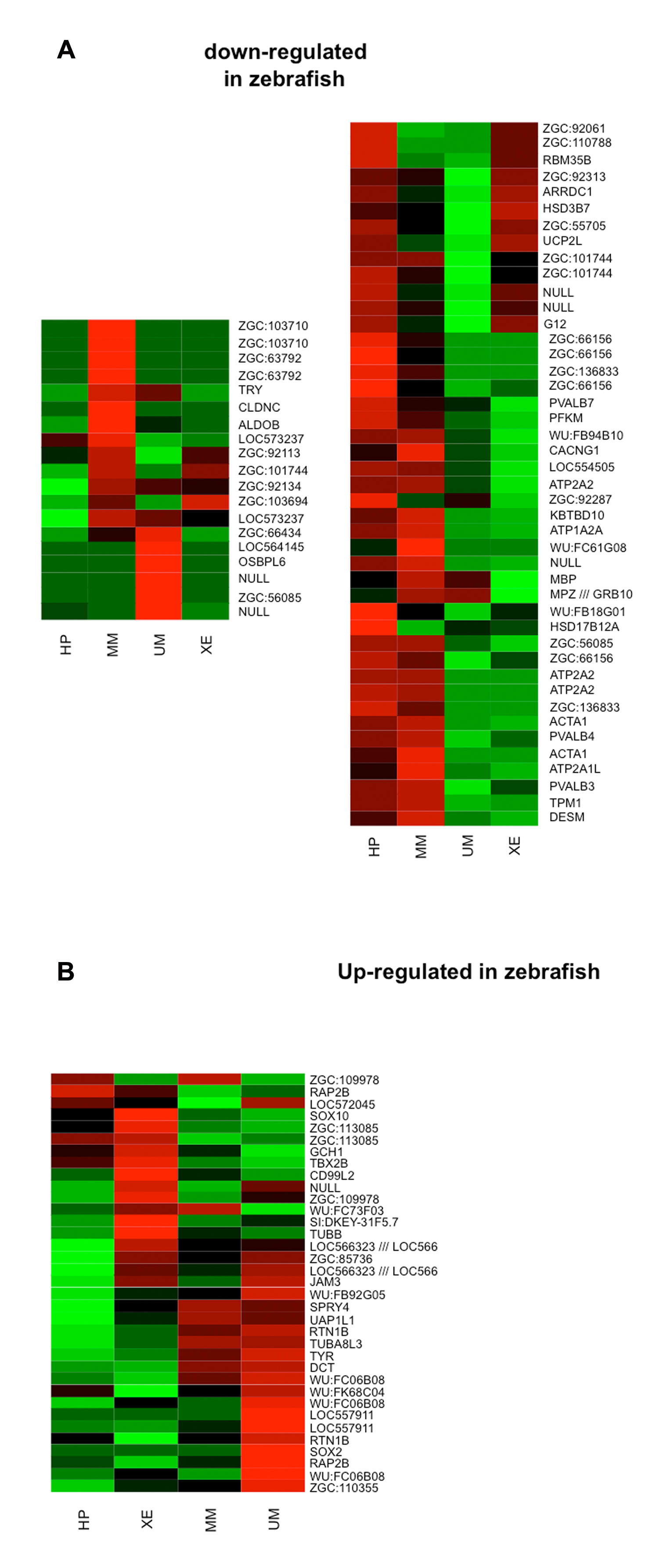

Supplement: Figure S3 — Heatplot (scaled) of genes common in zebrafish and medaka, displaying the quality of the log2 read count distribution within the genes. Low read counts are colored in green, high read counts are colored in red. 3a: Genes down-regulated in zebrafish, 3b: Genes up-regulated in zebrafish. (TIF) [file pone.0037880.s003.tif]

functional annotation  
Ease < 0.01, Count > 5

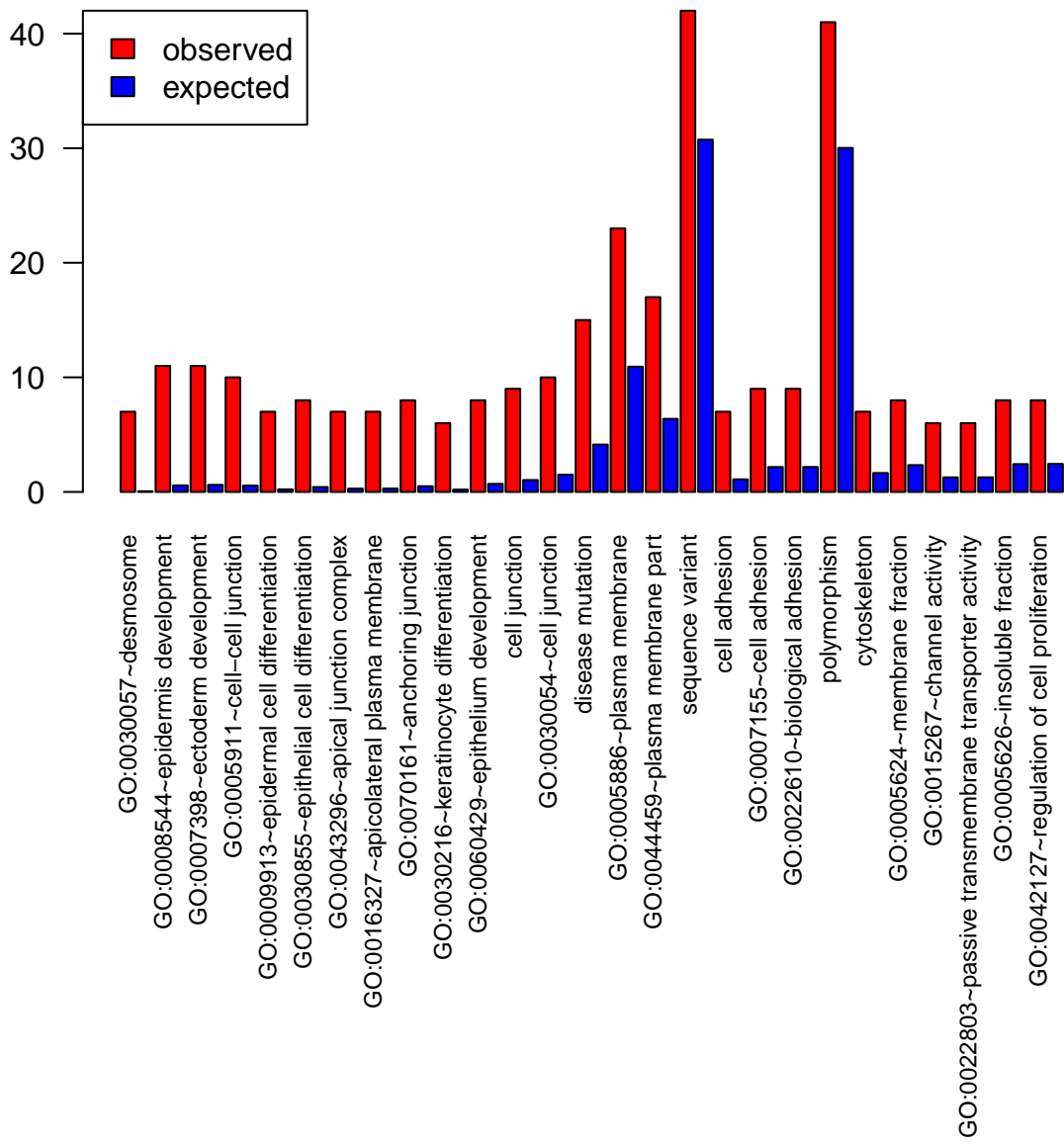

Supplement: Figure S4 — Affected pathways based on genes commonly upregulated or downregulated more than 2-fold in human cutaneous primary melanoma compared to melanocytic skin nevi and fish tumors (XE, UM and MM) compared to fish nevi (HP). Red bars show the number of observed genes up-regulated or down-regulated in the dataset, blue bars show the statistically expected number of genes, given the result to be random. (PDF) [file pone.0037880.s004.pdf]

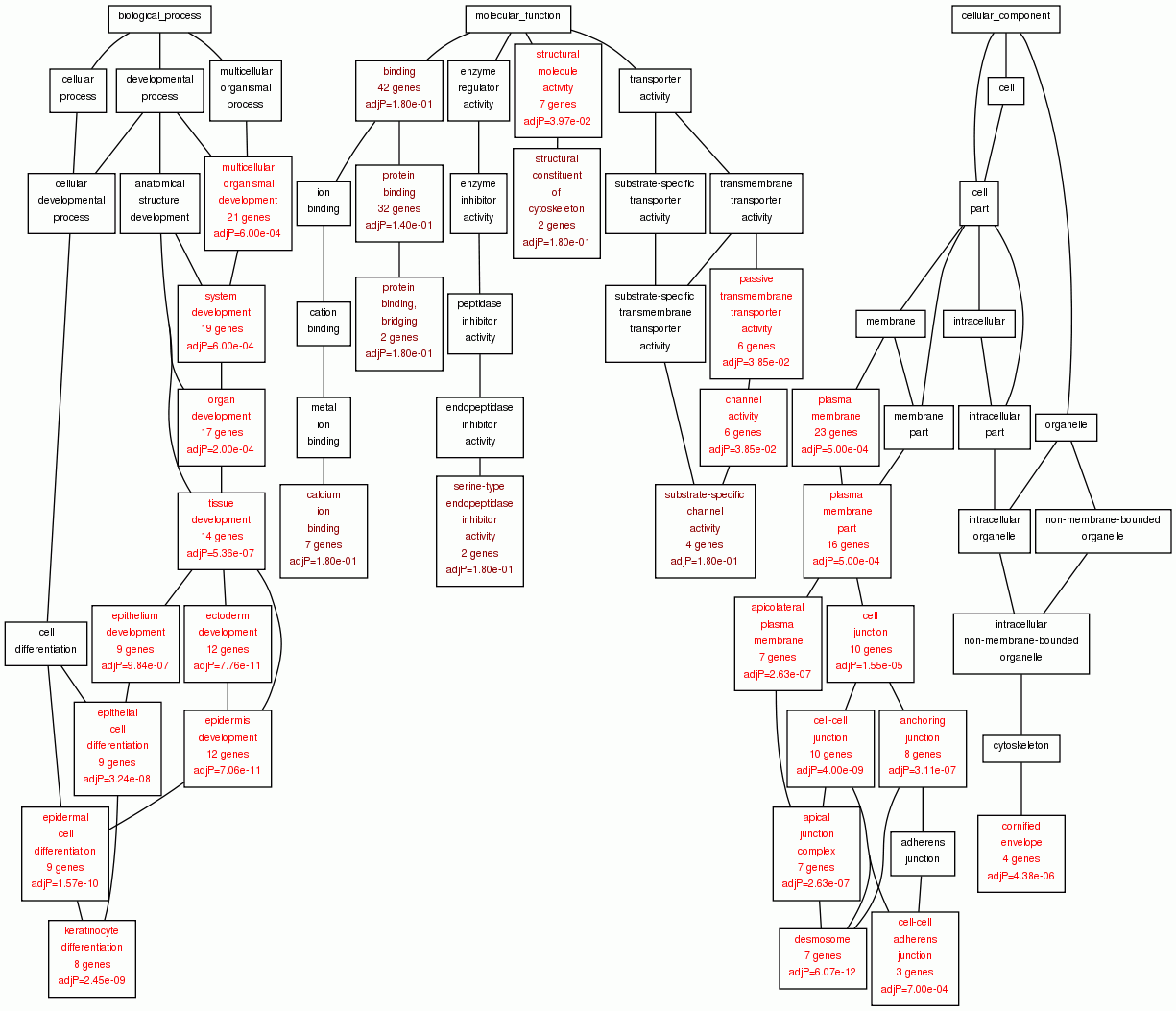

Supplement: Figure S5 — Gene Ontology analysis of functional gene groups commonly regulated in medaka tumors versus HP. The analysis was performed using the Gene Set Analysis Toolkit V2 (http://bioinfo.vanderbilt.edu/webgestalt/). (GIF) [file pone.0037880.s005.gif]
