# Supplementary material for: Conserved Expression Signatures between Medaka and Human Pigment Cell Tumors
Source: PLoS One. 2012 May 31;7(5):e37880. doi: 10.1371/journal.pone.0037880 (PMC3365055; doi:10.1371/journal.pone.0037880)
Supplement: Table S1 — Primers used for quantitative real-time PCR analysis. (DOC) [file pone.0037880.s006.doc]

**Supplementary Table 1:** Primers used for PCR amplification

| **Gene** | **Name** | **Sequence (5’ – 3’)** |
| --- | --- | --- |
| dickkopf homolog 3 (DKK 3) | Med-DKK3-1-for2 | AGGAAGGATGAGGAGTGCTG |
|  | Med-DKK3-1-rev2 | GGTTTGGGGTTACACACTGG |
| frizzled- related protein (FRZB) | Med-FRZB-for | CGTGGAACATGACCAAGATG |
|  | Med-FRZB-rev | CTGGAAATCAATGGTGCAGA |
| SOX10b | Med-SOX10b-for 2 | TGAGGTCACCCACTACAAGG |
|  | Med-SOX10b-rev 2 | CTCCATCCCTCTTCCCATC |
| N-Cadherin (1) | Ola_NCad1_up | TAGAGGCTGACGGAGTCGTT |
|  | Ola_NCad1_down | TCTTCATGCGTTTCAGTTCG |
| N-Cadherin (2) | Ola_NCad2_up | ATCTCAGGCCAGCTGTCAGT |
|  | Ola_NCad2_down | TTTGGACCCCTCAGGTACAG |
| β-actin | Hsa-actfor | GGCATCCTGACCCTGAAGTA |
|  | Hsa-actref | GGGGTGTTGAAGGTCTCAAA |
| SLC45A2 | Hsa-slcfor | AGAAGGGCCTCCACTACCAT |
|  | Hsa-sclrev | GTGAGCACCAATGCAGAGAA |
|  | Ola_SLC45A2-5`neu | GCCAAACAGCCAATTAAGGA |
|  | Ola_SLC45A2-3`neu | GCAGCCAACTTCAACTCCTC |
| Elongation factor-1 alpha | MF_ef1a1-f01 | GCCCCTGGACACAGAGACTTCATCA |
|  | MF_ef1a1-r01 | AAGGGGGCTCGGTGGAGTCCAT |

**Supplementary table 4:** Number of genes with RPKM > 2 showing an at least 2-fold up or down regulation in different tumor types compared to hyperpigmented skin

|  | XE vs. HP | MM vs. HP | UM vs. HP | Tumor vs. HP |
| --- | --- | --- | --- | --- |
| Up-regulated | 1263 (5.12%) | 1438 (5.83%) | 1901 (7.71%) | 2357 (9.56%) |
| Down-regulated | 1659 (6.73%) | 1488 (6.03%) | 2704 (10.96%) | 1046 (4.24%) |
| Total | 2922 (11.85%) | 2926 (11.86%) | 4605 (18.67%) | 3403 (13.80%) |
