# Supplementary material for: Conserved Expression Signatures between Medaka and Human Pigment Cell Tumors
Source: PLoS One. 2012 May 31;7(5):e37880. doi: 10.1371/journal.pone.0037880 (PMC3365055; doi:10.1371/journal.pone.0037880)
Supplement: Table S4 — Number of genes with RPKM>2 showing an at least 2-fold up or down regulation in different tumor types compared to hyperpigmented skin. (DOC) [file pone.0037880.s009.doc]

**Supplementary table 4:** Number of genes with RPKM > 2 showing an at least 2-fold up or down regulation in different tumor types compared to hyperpigmented skin

|  | XE vs. HP | MM vs. HP | UM vs. HP | Tumor vs. HP |
| --- | --- | --- | --- | --- |
| Up-regulated | 1263 (5.12%) | 1438 (5.83%) | 1901 (7.71%) | 2357 (9.56%) |
| Down-regulated | 1659 (6.73%) | 1488 (6.03%) | 2704 (10.96%) | 1046 (4.24%) |
| Total | 2922 (11.85%) | 2926 (11.86%) | 4605 (18.67%) | 3403 (13.80%) |
